# Prenatal Iron Deficiency and Replete Iron Status Are Associated with Adverse Birth Outcomes, but Associations Differ in Ghana and Malawi

**DOI:** 10.1093/jn/nxy278

**Published:** 2019-01-09

**Authors:** Brietta M Oaks, Josh M Jorgensen, Lacey M Baldiviez, Seth Adu-Afarwuah, Ken Maleta, Harriet Okronipa, John Sadalaki, Anna Lartey, Per Ashorn, Ulla Ashorn, Stephen Vosti, Lindsay H Allen, Kathryn G Dewey

**Affiliations:** 1 Department of Nutrition and Food Sciences, University of Rhode Island, Kingston, RI; 2 Program in International and Community Nutrition, Department of Nutrition; 3 Department of Agricultural and Resource Economics, University of California, Davis, CA; 4 USDA, Agricultural Research Service Western Human Nutrition Research Center, Davis, CA; 5 Department of Nutrition and Food Science, University of Ghana, Legon, Ghana; 6 Department of Community Health, University of Malawi College of Medicine, Blantyre, Malawi; 7 Department of Paediatrics, Tampere University Hospital, Tampere, Finland; 8 Center for Child Health Research, University of Tampere Faculty of Medicine and Life Sciences and Tampere University Hospital, Tampere, Finland

**Keywords:** pregnancy, iron deficiency, preterm birth, low birth weight, newborn stunting, anemia, iron status, Ghana, Malawi

## Abstract

**Background:**

Previous literature suggests a U-shaped relation between hemoglobin concentration and adverse birth outcomes. There is less evidence on associations between iron status and birth outcomes.

**Objective:**

Our objective was to determine the associations of maternal hemoglobin concentration and iron status with birth outcomes.

**Methods:**

We conducted a secondary data analysis of data from 2 cohorts of pregnant women receiving iron-containing nutritional supplements (20–60 mg ferrous sulfate) in Ghana (*n* = 1137) and Malawi (*n* = 1243). Hemoglobin concentration and 2 markers of iron status [zinc protoporphyrin and soluble transferrin receptor (sTfR)] were measured at ≤20 weeks and 36 weeks of gestation. We used linear and Poisson regression models and birth outcomes included preterm birth (PTB), newborn stunting, low birth weight (LBW), and small-for-gestational-age.

**Results:**

Prevalence of iron deficiency (sTfR >6.0 mg/L) at enrollment was 9% in Ghana and 20% in Malawi. In early pregnancy, iron deficiency was associated with PTB (9% compared with 17%, adjusted RR: 1.63; 95% CI: 1.14, 2.33) and stunting (15% compared with 23%, adjusted RR: 1.44; 95% CI: 1.09, 1.94) in Malawi but not Ghana, and was not associated with LBW in either country; replete iron status (sTfR <10th percentile) was associated with stunting (9% compared with 15%, adjusted RR: 1.71; 95% CI: 1.06, 2.77) in Ghana, but not PTB or LBW, and was not associated with any birth outcomes in Malawi. In late pregnancy, iron deficiency was not related to birth outcomes in either country and iron-replete status was associated with higher risk of LBW (8% compared with 16%, adjusted RR: 1.90; 95% CI: 1.17, 3.09) and stunting (6% compared with 13%, adjusted RR: 2.14; 95% CI: 1.21, 3.77) in Ghana, but was not associated with birth outcomes in Malawi.

**Conclusions:**

The associations of low or replete iron status with birth outcomes are population specific. Research to replicate and extend these findings would be beneficial. These trials were registered at clinicaltrials.gov as NCT00970866 (Ghana) and NCT01239693 (Malawi).

## Introduction

The WHO currently recommends daily supplementation with 30–60 mg/d elemental iron (+400 μg folic acid) throughout pregnancy, and in settings where anemia in pregnant women is a severe public health problem (prevalence of ≥40%), the daily dose of 60 mg is recommended over a lower dose (1). Although this amount of supplementation clearly reduces maternal iron deficiency and anemia, it is questionable whether there is a beneficial impact on birth outcomes (2) and there is concern that high maternal iron status may have a negative impact on the newborn (3).

Numerous studies have reported a U-shaped relation between maternal hemoglobin (Hb) concentrations during pregnancy and adverse birth outcomes, with both low and high Hb concentrations associated with higher risks of low birth weight (LBW) and preterm birth (PTB) (4–7), although it is unclear whether these associations are due to maternal iron status or other mechanisms. Research incorporating specific biomarkers of iron status is comparatively limited and has been conducted primarily in higher-income countries where women typically take lower doses of iron during pregnancy: increased risk of adverse birth outcomes has been observed with both low (8, 9) and high (10–14) maternal iron status.

A recent review noted that in studies that have examined maternal iron status and birth outcomes, serum ferritin concentration is the most widely used biomarker of iron status (15). Serum ferritin concentration is an indicator of iron stores, but is affected by inflammation. Furthermore, maternal plasma volume normally expands during pregnancy [mainly in the second and third trimesters (16, 17)] which leads to lower concentrations of Hb, ferritin, and other biomarkers. Thus, relatively high concentrations of Hb or ferritin in the second and third trimesters could be a marker of inadequate plasma volume expansion, which is associated with adverse birth outcomes. Soluble transferrin receptor (sTfR) and zinc protoporphyrin (ZPP) are alternative markers of iron status that may also be affected by inflammation, but because they respond to iron deficiency in the opposite direction to ferritin, lower values of sTfR or ZPP indicate higher iron status, which would not be caused by failure of plasma volume expansion.

Little research has been conducted regarding maternal iron status and birth outcomes in sub-Saharan Africa (15), despite the high prevalence of maternal anemia and iron deficiency in the region. The aim of this cross-country comparison was to examine the associations of maternal Hb concentration and iron status during pregnancy with birth outcomes in Ghana and Malawi. In particular, we were interested in both low and high Hb concentration and iron status, as well as whether the timing of these measurements (early compared with late pregnancy) had any influence on the aforementioned associations.

## Methods

### 

#### Participants and study design

Women included in this cohort study were from 2 randomized controlled trials conducted in Malawi (NCT01239693) and Ghana (NCT00970866), both malaria-endemic countries, as part of the International Lipid-Based Nutrient Supplements Project (www.ilins.org). The primary objective of these trials was to determine the efficacy of small-quantity lipid-based nutrient supplements (SQ-LNSs) for preventing malnutrition in pregnant and lactating women and their infants. Details of the study methods have been reported elsewhere (18–20). Briefly, the trials were similar in design, but each trial operated independently and there were some differences in inclusion and exclusion criteria. In both trials, women ≤20 weeks of gestation were randomly assigned to receive daily throughout pregnancy either: *1*) a 60-mg Fe (ferrous sulfate) +400-µg folic acid capsule; *2*) a multiple micronutrient capsule (18 micronutrients); or *3*) a sachet of SQ-LNS (118 kcal, 22 micronutrients, essential fatty acids, and protein). Both the multiple micronutrient capsule and SQ-LNS contained 20 mg Fe (ferrous sulfate) per daily supplement. Differences in inclusion and exclusion criteria included maternal age (Ghana trial: ≥18 y of age; Malawi trial: ≥15 y of age) and HIV status (Ghana trial: excluded women if they were HIV positive; Malawi trial: did not exclude women if they were HIV positive). Women received intermittent preventive malaria treatment in accordance with standard antenatal care recommendations in Ghana and Malawi. At both enrollment and 36 weeks of gestation, trained phlebotomists collected blood samples by venipuncture with heparinized blood collection tubes. Blood samples were typically collected in the morning, with the majority of samples collected between 0800 and 1200. Previous studies suggest that time of day does not affect the reliability of markers of iron status (21). In Ghana, we referred any woman with Hb <70 g/L at baseline or <100 g/L at 36 weeks of gestation to the hospital for treatment of anemia, but allowed them to remain in the study. In Malawi, we excluded women with Hb <50 g/L at baseline (<0.1% of women) and referred them for treatment; women with Hb 50–69 g/L at baseline were treated with iron supplements if found to be iron deficient according to ZPP plasma concentration and were allowed to remain in the study. Both trials collected sociodemographic information and trained anthropometrists measured maternal weight and height at enrollment.

Women from these trials were included in this cohort study if they had: *1*) at least one measurement of Hb concentration or iron status, at either enrollment or 36 weeks of gestation, and *2*) any newborn anthropometric measurement or an estimated pregnancy duration. We have previously reported that supplementation with SQ-LNSs or multiple micronutrients resulted in lower Hb and iron status at 36 weeks of gestation than for the iron and folic acid trial arm (22, 23). Assigned supplement group in the main trial was not the focus of the current study and was controlled for in analyses of the 36-wk measurements.

#### Birth outcomes

In Ghana, newborn measurements were obtained by fieldworkers within 48 h after birth for 90.6% of infants and between 3 and 14 d for 9.4% of infants. In Malawi, fieldworkers measured birth weight usually within 48 h after birth, with 10.9% of birth weights obtained between 3 and 14 d. All other newborn anthropometry for the Malawi trial was completed at a clinic visit 1–2 wk after birth. All newborn anthropometric measurements obtained 3–14 d after birth were back-calculated using formulae described by the WHO (24). Fieldworkers measured birth weight to the nearest 20 g (Seca 383; Seca GmbH & Co.), length to the nearest 0.1 cm (Ghana: Seca 416; Seca GmbH & Co.; Malawi: Harpenden Infantometer; Holtain Limited), and head circumference to the nearest 0.1 cm (Shorrtape, Weight and Measure, LLC). We determined gestational age at enrollment by ultrasound (Ghana: Aloka SSD 500; Malawi: EDAN DUS 3 Digital Ultrasonic Diagnostic Imaging System; EDAN Instruments Inc.) and date of birth was obtained by interviewing the mother and confirmed with the infant's hospital or health card when available.

#### Laboratory analysis

Laboratory technicians assayed Hb (HemoCue) and malaria parasitemia with rapid tests (Ghana: Vision Biotech; Malawi: Clearview Malaria Combo; British Biocell International Ltd.) in peripheral blood and centrifuged the remaining blood samples at 1252 × *g* for 15 min at room temperature to separate RBCs and plasma. RBCs were washed 3 times with normal saline and we used the original Aviv cover-slides and 3-level control material for the ZPP measurements obtained using a hematofluorometer (Aviv Biomedical, Inc.). Plasma samples were stored at −20°C and shipped to the USDA Agricultural Research Service Western Human Nutrition Research Center, Davis, CA where sTfR (mg/L), α-1-acid glycoprotein [AGP (g/L)], and C-reactive protein [CRP (mg/L)] concentrations were determined using a Cobas Integra 400 plus Automatic Analyzer (Roche Diagnostic Corp.). The inter- and intra-assay CVs for these assays were: *1*) sTfR intra-assay: <2.2%, interassay: <1.1%; *2*) AGP intra-assay: <2.9%, interassay: <2.0%; *3*) CRP: intra-assay: <3.5%, interassay: <1.3%.

#### Statistical analysis

We tested normality using the Shapiro–Wilk test. Hb was determined to have a normal distribution and ZPP and sTfR concentrations were log transformed. We also analyzed Hb, ZPP, and sTfR using categorical variables. We defined anemia using a cutoff value of Hb <100 g/L, based on research suggesting that this cutoff value is more accurate when defining anemia in pregnant women of African descent (7, 25, 26). We defined high Hb as >130 g/L (2) and elevated sTfR (proxy for tissue iron deficiency) as >6.0 mg/L. We derived the 6.0 mg/L cutoff value for sTfR based on previous research reporting that sTfR values obtained using the Automatic Analyzer assay (as used in this study) are on average 30% lower than values obtained with the ELISA assay (27). Therefore, we reduced by 30% the 8.5 mg/L cutoff value used when sTfR is determined with ELISA (28) to obtain the cutoff of ∼6.0 mg/L for our analysis. We defined iron deficiency anemia (IDA) as Hb <100 g/L and sTfR >6.0 mg/L. A study in an adult nonpregnant African population indicated that sTfR concentration is inversely correlated with tissue iron concentrations and is decreased in the presence of iron overload (29), although there is no such evidence for ZPP. Due to the lack of a published cutoff for low sTfR, we categorized women as iron replete if sTfR was <10th percentile based on the distribution within each cohort.

For each country, we used linear regression models to examine the associations of Hb, ZPP, and sTfR with birth outcomes as continuous variables [duration of gestation, birth weight, length-for-age *z* score (LAZ), and head circumference *z* score (HCZ)]. Poisson regression models were used to estimate RR for dichotomous birth outcomes, including PTB (<37 weeks of gestation), LBW (<2.5 kg), small-for-gestational-age (SGA) [birth weight <10th percentile by gestational age and sex using the INTERGROWTH-21^st^ standard (30, 31)], and stunting (LAZ <−2), in association with the dichotomous predictors of Hb and iron status defined previously. PTB was examined only with respect to measurements of Hb and iron status taken at enrollment because many PTBs occurred before the 36 wk blood draw. HCZ was not analyzed as a categorical variable owing to a low number of infants with HCZ <−2. We checked all models for U-shaped relations using quadratic terms and found a lack of any U-shaped relations except for 1 association (Hb concentration at 36 wk and pregnancy duration). We considered covariates for inclusion in the model if they were significantly (*P* < 0.1) associated with the outcome in bivariate analyses. Based on previous literature, variables identified a priori as potential confounding factors were gestational age at enrollment, parity (nulliparous compared with parous), maternal age, education level, household food insecurity, household asset index, AGP at the time the blood sample was drawn, CRP at the time the blood sample was drawn, infant sex, baseline maternal BMI (in kg/m^2^), a positive rapid test for malaria at enrollment, and HIV status (Malawi models only). Household food insecurity was measured using a scale ranging from 0 (no food insecurity) to 27 (every food insecurity condition occurs often) (32). We created the household asset index based on lighting source, drinking water supply, sanitation facilities, flooring materials, radio, television, refrigerator, cell phone, and stove using principal components analysis (33). Specific multivariable models for each outcome are provided in the **Supplemental Methods**. All multivariable models examining 36-wk measurements of Hb and iron status were also adjusted for intervention group.

Interactions with maternal age, parity, and supplement group were examined and determined to be significant at *P* < 0.1, and stratified analyses were performed for significant interactions. All analyses were performed using SAS version 9.4 (SAS Institute).

## Results

Of the 1320 and 1391 women enrolled in the trials in Ghana and Malawi, respectively, 1137 and 1243 were included in this set of analyses, respectively. Reasons for exclusion included twin pregnancy (Ghana: *n* = 22, Malawi: *n* = 12), miscarriage (Ghana: *n* = 37, Malawi: *n* = 10), stillbirth (Ghana: *n* = 29, Malawi: *n* = 26), and loss-to-follow-up (Ghana: *n* = 95, Malawi: *n* = 100) (**Supplemental Figure 1**). In Malawi, included participants were more likely to be older (25 compared with 24 y, *P* < 0.001); have a lower proxy SES (−0.04 compared with 0.18, *P* < 0.002); and be less often nulliparous (19.7% compared with 28.8%, *P* < 0.001), HIV positive (12.5% compared with 17.8%, *P* = 0.029), or anemic (18.6% compared with 27.6%, *P* < 0.001) than excluded participants, but they had similar BMI, years of education, and malaria rates at enrollment to those excluded. In Ghana, excluded study participants did not differ from included participants in any of the aforementioned listed characteristics. Study participant characteristics are provided in **Table 1**.

**TABLE 1:** Characteristics of 2 study cohorts of pregnant women in Ghana and Malawi at ≤20 weeks of gestation^1^

	Ghana (*n* = 1137)	Malawi (*n* = 1243)	*P*
Maternal age, y	26.7 ± 5.5	25.3 ± 6.1	<0.001
Nulliparous women	33.8	19.7	<0.001
Male fetus^2^	48.4	48.7	0.88
Gestational age, wk	16.1 ± 3.3	16.8 ± 2.1	<0.001
BMI, kg/m^2^	24.7 ± 4.2	22.1 ± 2.8	<0.001
Overweight or obese (BMI ≥ 25 kg/m^2^)	42.3	12.0	<0.001
Low BMI (<18.5 kg/m^2^)	2.9	5.6	0.001
Positive HIV test	—^3^	12.5	—^3^
Positive malaria test	10.2	22.7	<0.001
Household Food Insecurity Index^4^	2.6 ± 4.3	4.9 ± 4.5	<0.001

^1^Values are means ± SDs or percentages; *P* values are for chi-square tests (categorical variables) or *t* tests (continuous variables).

^2^Based on infant sex at birth.

^3^HIV+ women were not enrolled in the Ghana trial.

^4^Scale ranging from 0 (no food insecurity) to 27 (every food insecurity condition occurs often).

Prevalence of anemia (Hb <100 g/L), iron deficiency (sTfR >6.0 mg/L), and IDA and mean concentrations of Hb, sTfR, and ZPP at enrollment and 36 weeks of gestation are presented in **Table 2**. Mean ± SD pregnancy duration was 39.3 ± 1.9 wk and 39.1 ± 2.9 wk in Ghana and Malawi, respectively. Newborn mean ± SD birth weight was 2982 ± 432 g in Ghana and 2970 ± 447 g in Malawi. Adverse birth outcomes included PTB (Ghana: 8.5%, Malawi: 10.0%), LBW (Ghana: 11.8%, Malawi: 12.8%), SGA (Ghana: 21.8%, Malawi: 29.5%), and stunting (Ghana: 9.4%, Malawi: 16.0%).

**TABLE 2:** Prevalence of anemia and iron deficiency and mean concentrations of Hb, markers of iron status, and inflammation at ≤20 weeks and 36 weeks of gestation in pregnant women in Ghana and Malawi^1^

	≤20 weeks of gestation			36 weeks of gestation		
	Ghana (*n* = 1137)^2^	Malawi (*n* = 1243)^2^	*P*	Ghana (*n* = 1137)^2^	Malawi (*n* = 1243)^2^	*P*
CRP, mg/L	6.9 ± 11.6	8.7 ± 17.8	0.003	5.7 ± 15.8	6.5 ± 14.1	0.27
AGP, g/L	0.65 ± 0.21	0.73 ± 0.25	<0.001	0.48 ± 0.20	0.56 ± 0.23	<0.001
Hb, g/L	111 ± 12	112 ± 16	0.85	117 ± 12	111 ± 15	<0.001
Anemia (Hb <100 g/L)	14.0	18.6	<0.001	6.0	19.6	<0.001
sTfR, mg/L	4.1 ± 2.6	4.7 ± 2.7	<0.001	4.5 ± 1.7	5.6 ± 3.0	<0.001
ZPP, µmol/mol heme	45 ± 28	53 ± 40	<0.001	46 ± 23	60 ± 41	<0.001
Iron deficiency (sTfR >6.0 mg/L)	9.3	19.7	<0.001	14.6	33.5	<0.001
IDA (Hb <100 g/L and sTfR >6.0 mg/L)	4.3	6.7	0.001	2.9	10.1	<0.001

^1^Values are means ± SDs or percentages; *P* values are for chi-square tests (categorical variables) or *t* tests (continuous variables). AGP, α-1-acid glycoprotein; CRP, C-reactive protein; Hb, hemoglobin; IDA, iron deficiency anemia; sTfR, soluble transferrin receptor; ZPP, zinc protoporphyrin.

^2^Missing data at enrollment for Ghana included CRP (*n* = 21), AGP (*n* = 21), sTfR (*n* = 21), ZPP (*n* = 2), and IDA (*n* = 21) and for Malawi included CRP (*n* = 7), AGP (*n* = 7), Hb (*n* = 1), sTfR (*n* = 7), ZPP (*n* = 52), and IDA (*n* = 7). Missing data at week 36 for Ghana included CRP (*n* = 156), AGP (*n* = 156), Hb (*n* = 152), sTfR (*n* = 156), ZPP (*n* = 157), and IDA (*n* = 159) and for Malawi included CRP (*n* = 140), AGP (*n* = 136), Hb (*n* = 165), sTfR (*n* = 136), ZPP (*n* = 187), and IDA (*n* = 183).

### 

#### Hb and iron status at enrollment—associations with birth outcomes

At enrollment, Hb concentration in adjusted linear regression analyses was not associated with any birth outcomes in Ghana but was associated with a longer pregnancy duration, higher birth weight, and higher newborn LAZ in Malawi (**Table 3**). Higher sTfR, indicating a lower iron status, was associated with a shorter pregnancy duration, lower birth weight, lower newborn LAZ, and lower HCZ in Malawi, but was associated with a higher newborn LAZ in Ghana. Higher ZPP, also an indicator of lower iron status, was associated with a shorter pregnancy duration and lower newborn LAZ in Malawi but was not associated with any birth outcomes in Ghana. **Supplemental Table 1** provides a nonnumeric visual overview of the associations presented in Table 3 and unadjusted results are presented in **Supplemental Table 2**. There were no significant interactions of Hb concentration or iron status at enrollment with maternal age, parity, or supplement group.

**TABLE 3:** Standardized regression coefficients of Hb, sTfR, and ZPP with pregnancy duration and newborn anthropometric indicators in Ghana and Malawi^1^

	Hb		sTfR		ZPP	
	Adjusted β (95% CI)^2^	*P*	Adjusted β (95% CI)^2^	*P*	Adjusted β (95% CI)^2^	*P*
Early pregnancy: <20 wk						
Pregnancy duration						
Ghana	0.05 (−0.01, 0.12)	0.11	0.00 (−0.06, 0.06)	0.92	0.04 (−0.02, 0.10)	0.18
Malawi	0.09 (0.03, 0.14)	0.004	−0.07, (−0.13, −0.02)	0.01	−0.07 (−0.12, −0.01)	0.02
Birth weight						
Ghana	0.03 (−0.03, 0.08)	0.39	0.02 (−0.04, 0.08)	0.48	0.01 (−0.04, 0.07)	0.68
Malawi	0.08 (0.02, 0.15)	0.006	−0.10 (−0.16, −0.04)	<0.001	−0.03 (−0.09, 0.03)	0.35
Newborn LAZ						
Ghana	0.01 (−0.05, 0.07)	0.66	0.06 (0.00, 0.12)	0.03	0.05 (0.00, 0.11)	0.07
Malawi	0.08 (0.02, 0.14)	0.007	−0.10 (−0.16, −0.04)	<0.001	−0.06 (−0.12, −0.003)	0.04
Newborn HCZ						
Ghana	0.03 (−0.03, 0.09)	0.35	0.03 (−0.03, 0.09)	0.37	0.04 (−0.02, 0.10)	0.21
Malawi	0.05 (−0.01, 0.11)	0.13	−0.07, (−0.13, −0.01)	0.03	−0.02 (−0.08, 0.04)	0.58
Late pregnancy: 36 wk						
Pregnancy duration						
Ghana	—^3^	—^3^	0.05 (−0.01, 0.09)	0.10	0.00 (−0.05, 0.04)	0.88
Malawi	−0.02 (−0.05, 0.01)	0.27	−0.03 (−0.07, 0.001)	0.055	−0.03 (−0.06, 0.002)	0.07
Birth weight						
Ghana	−0.04 (−0.10, 0.02)	0.18	0.10 (0.03, 0.15)	0.002	0.09 (0.02, 0.14)	0.01
Malawi	−0.03 (−0.08, 0.03)	0.32	0.001 (−0.06, 0.06)	0.96	0.04 (−0.02, 0.09)	0.24
Newborn LAZ						
Ghana	−0.05 (−0.10, 0.01)	0.12	0.10 (0.03, 0.15)	0.002	0.07 (0.00, 0.12)	0.04
Malawi	0.001 (−0.05, 0.06)	0.97	0.01 (−0.05, 0.06)	0.85	0.01 (−0.04, 0.07)	0.69
Newborn HCZ						
Ghana	−0.03 (−0.09, 0.03)	0.33	0.07 (0.01, 0.12)	0.03	0.04 (−0.02, 0.09)	0.18
Malawi	−0.05 (−0.10, 0.01)	0.14	0.04 (−0.02, 0.09)	0.21	0.07 (−0.0004, 0.12)	0.052

^1^Hb, hemoglobin; HCZ, head-circumference-for-age *z* score; LAZ, length-for-age *z* score; sTfR, soluble transferrin receptor; ZPP, zinc protoporphyrin.

^2^Standardized regression coefficients. Adjusted models included the following covariates if significantly (*P* < 0.1) associated with the outcome: gestational age at enrollment, parity, maternal age, education level, household food insecurity, household asset index, α-1-acid glycoprotein at the time the blood sample was drawn, C-reactive protein at the time the blood sample was drawn, infant sex, maternal BMI at enrollment, maternal malaria at enrollment, and HIV status (Malawi models only). All 36-wk models adjusted for intervention group. Specifics regarding each adjusted model are provided in the Supplemental Methods.

**FIGURE 1:**
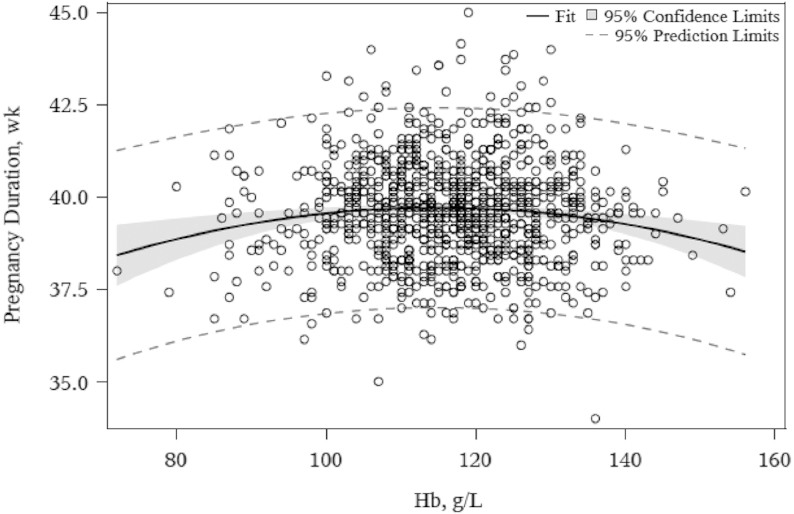
Association between pregnancy duration and maternal Hb concentration measured at 36 weeks of gestation in Ghanaian pregnant women (*n* = 985). Hb, hemoglobin.

^3^U-shaped relation; see Figure 1.

Anemia (**Supplemental Table 3**), iron deficiency (**Table 4**), and IDA (**Table 5**) at enrollment were all associated with an increased risk of PTB in Malawi; iron deficiency and IDA were also associated with increased risk of newborn stunting. In Ghana, anemia was not associated with adverse birth outcomes; however, IDA was associated with a decreased risk of LBW (*P* = 0.046) and iron deficiency showed a similar trend (*P* = 0.07). High Hb (>130 g/L) was not associated with adverse birth outcomes in either Ghana or Malawi (**Supplemental Table 4**), although replete iron status (sTfR <10th percentile) was associated with an increased risk of newborn stunting in Ghana (**Table 6**). Unadjusted results for anemia, iron deficiency, IDA, high Hb, and iron-replete status are presented in **Supplemental Tables 5–9**.

**TABLE 4:** Risk of adverse birth outcomes for women with iron deficiency during early or late pregnancy^1^

	Without iron deficiency, *n*/total *n* (%)^2^	With iron deficiency, *n*/total *n* (%)^3^	Adjusted RR (95% CI)^4^	*P*
Early pregnancy: ≤20 wk				
PTB				
Ghana	77/924 (8.3)	10/102 (9.8)	1.13 (0.61, 2.11)	0.70
Malawi	82/912 (9.0)	41/248 (16.5)	1.63 (1.14, 2.33)	0.007
LBW				
Ghana	110/923 (11.9)	7/102 (6.9)	0.51 (0.24, 1.05)	0.07
Malawi	100/813 (12.3)	35/210 (16.7)	1.24 (0.87, 1.75)	0.23
SGA				
Ghana	194/889 (21.8)	19/100 (19.0)	0.87 (0.57, 1.33)	0.53
Malawi	195/788 (24.7)	58/205 (28.3)	1.15 (0.89, 1.49)	0.27
Newborn stunting				
Ghana	78/918 (8.5)	7/102 (6.9)	0.81 (0.39, 1.68)	0.56
Malawi	114/762 (15.0)	48/205 (23.4)	1.44 (1.09, 1.94)	0.01
Late pregnancy: 36 wk				
LBW				
Ghana	58/753 (7.7)	10/141 (7.1)	0.78 (0.42, 1.44)	0.43
Malawi	53/560 (9.5)	34/327 (10.4)	0.97 (0.65, 1.46)	0.89
SGA				
Ghana	161/735 (21.9)	28/138 (20.3)	0.82 (0.57, 1.18)	0.28
Malawi	137/546 (25.1)	84/321 (26.2)	1.03 (0.82, 1.29)	0.81
Newborn stunting				
Ghana	42/750 (5.6)	6/141 (4.3)	0.64 (0.28, 1.45)	0.28
Malawi	76/536 (14.2)	38/317 (12.0)	0.76 (0.53, 1.08)	0.13

^1^PTB: <37 weeks of gestation; LBW: <2.5 kg; SGA: birth weight <10th percentile by gestational age and sex using the INTERGROWTH-21^st^ standard (30); stunting: length-for-age *z* score <−2. LBW, low birth weight; PTB, preterm birth; SGA, small-for-gestational-age; sTfR, soluble transferrin receptor.

^2^Women with sTfR ≤6.0 mg/L. The reference group excluded women with iron-replete status (sTfR <10th percentile). At ≤20 wk, this was <2.49 mg/L for Ghana and <2.65 mg/L for Malawi. At 36 wk, this was <2.86 mg/L for Ghana and <3.08 mg/L for Malawi.

^3^sTfR >6 mg/L.

^4^Adjusted models included the following covariates if significantly (*P* < 0.1) associated with the outcome: gestational age at enrollment, parity, maternal age, education level, household food insecurity, household asset index, α-1-acid glycoprotein at the time the blood sample was drawn, C-reactive protein at the time the blood sample was drawn, infant sex, maternal BMI at enrollment, maternal malaria at enrollment, and HIV status (Malawi models only). All 36-wk models adjusted for intervention group. Specifics regarding each adjusted model are provided in the Supplemental Methods.

**TABLE 5:** Risk of adverse birth outcomes for women with IDA during early or late pregnancy^1^

	Without IDA, *n*/total *n* (%)^2^	With IDA, *n*/total *n* (%)^3^	Adjusted RR (95% CI)^4^	*P*
Early pregnancy: ≤20 wk				
PTB				
Ghana	92/1089 (8.5)	5/48 (10.4)	1.13 (0.52, 2.47)	0.75
Malawi	104/1181 (8.8)	25/106 (23.6)	2.23 (1.48, 3.37)	<0.001
LBW				
Ghana	132/1088 (12.1)	1/48 (2.1)	0.14 (0.02, 0.97)	0.046
Malawi	129/1051 (12.3)	17/86 (19.8)	1.45 (0.94, 2.25)	0.10
SGA				
Ghana	234/1051 (21.3)	6/48 (12.5)	0.55 (0.26, 1.17)	0.12
Malawi	306/1051 (29.1)	26/83 (31.3)	1.38 (0.99, 1.91)	0.057
Newborn stunting				
Ghana	101/1083 (9.3)	1/48 (2.1)	0.21 (0.03, 1.45)	0.11
Malawi	147/997 (14.7)	25/85 (29.4)	1.85 (1.30, 2.64)	<0.001
Late pregnancy: 36 wk				
LBW				
Ghana	81/963 (8.4)	2/27 (7.4)	0.57 (0.18, 1.80)	0.34
Malawi	86/837 (10.3)	10/102 (9.8)	0.85 (0.46, 1.57)	0.60
SGA				
Ghana	217/941 (23.1)	5/27 (18.5)	0.57 (0.22, 1.43)	0.23
Malawi	251/837 (30.0)	25/101 (24.8)	0.98 (0.70, 1.38)	0.92
Newborn stunting				
Ghana	57/960 (5.9)	2/27 (7.4)	0.73 (0.18, 2.94)	0.66
Malawi	105/810 (13.0)	16/95 (16.8)	1.19 (0.76, 1.86)	0.45

^1^PTB: <37 weeks of gestation; LBW: <2.5 kg; SGA: birth weight <10th percentile by gestational age and sex using the INTERGROWTH-21^st^ standard (30); stunting: length-for-age *z* score <−2. Hb, hemoglobin; IDA, iron deficiency anemia; LBW, low birth weight; PTB, preterm birth; SGA, small-for-gestational-age; sTfR, soluble transferrin receptor.

^2^Women with Hb ≥100 g/L and sTfR ≤6.0 mg/L.

^3^Defined as Hb <100 g/L and sTfR >6.0 mg/L.

^4^Adjusted models included the following covariates if significantly (*P* < 0.1) associated with the outcome: gestational age at enrollment, parity, maternal age, education level, household food insecurity, household asset index, α-1-acid glycoprotein at the time the blood sample was drawn, C-reactive protein at the time the blood sample was drawn, infant sex, maternal BMI at enrollment, maternal malaria at enrollment, and HIV status (Malawi models only). All 36-wk models adjusted for intervention group. Specifics regarding each adjusted model are provided in the Supplemental Methods.

**TABLE 6:** Risk of adverse birth outcomes for women with iron-replete status during early or late pregnancy^1^

	Reference group (sTfR ≥10th percentile), *n*/total *n* (%)^2^	With iron-replete status (sTfR <10th percentile), *n*/total *n* (%)^3^	Adjusted RR (95% CI)^4^	*P*
Early pregnancy: ≤20 wk				
PTB				
Ghana	77/924 (8.3)	10/111 (9.0)	0.88 (0.44, 1.76)	0.71
Malawi	82/912 (9.0)	6/128 (4.7)	0.66 (0.29, 1.48)	0.31
LBW				
Ghana	110/923 (11.9)	16/111 (14.4)	1.06 (0.66, 1.70)	0.81
Malawi	100/813 (12.3)	11/115 (9.6)	0.81 (0.46, 1.45)	0.80
SGA				
Ghana	194/889 (21.8)	27/110 (24.6)	1.09 (0.76, 1.55)	0.64
Malawi	195/788 (24.7)	24/111 (21.6)	0.82 (0.57, 1.19)	0.30
Newborn stunting				
Ghana	78/918 (8.5)	17/111 (15.3)	1.71 (1.06, 2.77)	0.03
Malawi	114/762 (15.0)	10/116 (8.6)	0.60 (0.33, 1.11)	0.10
Late pregnancy: 36 wk				
LBW				
Ghana	58/753 (7.7)	16/99 (16.2)	1.90 (1.17, 3.09)	0.01
Malawi	53/560 (9.5)	14/99 (14.1)	1.39 (0.81, 2.39)	0.23
SGA				
Ghana	161/735 (21.9)	34/98 (34.7)	1.51 (1.12, 2.05)	0.01
Malawi	137/546 (25.1)	23/98 (23.5)	0.92 (0.62, 1.36)	0.67
Newborn stunting				
Ghana	42/750 (5.6)	13/99 (13.1)	2.14 (1.21, 3.77)	0.01
Malawi	76/536 (14.2)	12/101 (11.9)	0.73 (0.42, 1.26)	0.26

^1^PTB: <37 weeks of gestation; LBW: <2.5 kg; SGA: birth weight <10th percentile by gestational age and sex using the INTERGROWTH-21^st^ standard (30); stunting: length-for-age *z* score <−2. LBW, low birth weight; PTB, preterm birth; SGA, small-for-gestational-age; sTfR, soluble transferrin receptor.

^2^The reference group excluded women with iron deficiency (sTfR >6.0 mg/L).

^3^sTfR <10th percentile; at ≤20 wk, this was <2.49 mg/L for Ghana and <2.65 mg/L for Malawi. At 36 wk, this was <2.86 mg/L for Ghana and <3.08 mg/L for Malawi.

^4^Adjusted models included the following covariates if significantly (*P* < 0.1) associated with the outcome: gestational age at enrollment, parity, maternal age, education level, household food insecurity, household asset index, α-1-acid glycoprotein at the time the blood sample was drawn, C-reactive protein at the time the blood sample was drawn, infant sex, maternal BMI at enrollment, maternal malaria at enrollment, and HIV status (Malawi models only). All 36-wk models adjusted for intervention group. Specifics regarding each adjusted model are provided in the Supplemental Methods.

In unadjusted analyses, there was a significantly higher risk of LBW for women with IDA in Malawi that was attenuated and became nonsignificant in adjusted results. All other unadjusted results were similar to adjusted results.

#### Hb and iron status at 36 weeks of gestation—associations with birth outcomes

At 36 weeks of gestation, a U-shaped relation between Hb concentration and pregnancy duration was evident in Ghana, with both low and high Hb concentrations associated with a shorter duration of gestation (**Figure 1**). Hb concentration was not associated with any other birth outcomes. Higher sTfR (indicator of lower iron status) was associated with a higher birth weight, LAZ, and HCZ in Ghana but was not associated with any birth outcomes in Malawi. Higher ZPP (indicator of lower iron status) was associated with a greater birth weight and LAZ in Ghana but was not associated with any birth outcomes in Malawi (Table 3). Supplemental Table 1 provides a nonnumeric visual overview of the associations presented in Table 3. Unadjusted results were similar to adjusted results and are presented in Supplemental Table 2. There were no significant interactions of Hb concentration or iron status at 36 wk with maternal age, parity, or supplement group.

Anemia (Supplemental Table 3), iron deficiency (Table 4), and IDA (Table 5) at 36 wk were not associated with any adverse birth outcomes in Ghana or Malawi. There were also no associations in either country between high Hb and adverse birth outcomes (Supplemental Table 4). Replete iron status was not associated with adverse birth outcomes in Malawi. In Ghana, replete iron status was associated with an increased risk of LBW, SGA, and newborn stunting (Table 6). Unadjusted results for anemia, iron deficiency, IDA, high Hb, and iron-replete status are presented in Supplemental Tables 5–9. All unadjusted results were similar to adjusted results.

## Discussion

This study examined associations of maternal Hb concentration and iron status with birth outcomes in 2 cohorts of pregnant women in Africa. In Malawi but not in Ghana, iron deficiency and IDA in early pregnancy were related to PTB and newborn stunting, and anemia in early pregnancy was also related to PTB. By contrast, in Ghana iron-replete status in early pregnancy was associated with newborn stunting, and iron deficiency and IDA in early pregnancy were related to lower risk of LBW. Anemia, iron deficiency, and IDA in late pregnancy were not significantly related to birth outcomes in either country, although replete iron status in late pregnancy was related to higher risk of LBW, SGA, and newborn stunting in Ghana.

In populations at risk of anemia or iron deficiency, higher maternal Hb concentrations and iron status have often been associated with better birth outcomes (34, 35). Higher Hb or higher iron status may improve the systemic response to inflammation and infection, reduce the stress response from chronic hypoxia, and lower oxidative stress via less erythrocyte oxidation (36, 37). However, previous studies did not distinguish anemia or iron deficiency by timing in pregnancy (i.e., early compared with late pregnancy). Associations with biomarkers in late pregnancy are complicated by the issue of plasma volume expansion (PVE). Maternal plasma volume expands during normal pregnancy, resulting in lower concentrations of Hb and other biomarkers. In iron-replete populations, higher Hb concentration and iron status have been associated with adverse birth outcomes (15); however, inadequate PVE is also associated with adverse birth outcomes, thus making it difficult to interpret associations of adverse birth outcomes with high Hb or ferritin (a marker of iron storage).

In Ghana and Malawi, we measured sTfR and ZPP concentrations as biomarkers of iron status. Ferritin was not included because its utility is limited during periods when there is little iron storage because of high requirements, such as the second and third trimesters of pregnancy. During such times, iron storage may be low but iron concentrations for physiological needs may still be adequate and may be more accurately assessed using other markers (38). Unlike Hb or ferritin, *lower* sTfR and ZPP concentrations indicate higher iron status. We did not measure PVE in our cohorts and it is possible that some women were experiencing inadequate PVE, which would have concentrated the biomarkers. As we did not have any significant associations with Hb >130 g/dL, and PVE would have biased the associations of sTfR and ZPP towards the null, we find it unlikely that inadequate PVE explains the associations between higher iron status (lower sTfR and ZPP concentrations) and poorer birth outcomes in Ghana. Lower sTfR concentration can be a sign of impaired RBC production (which is linked to inadequate PVE) (39) and possibly would have biased the results away from the null. However, we find it unlikely that impaired RBC production would occur more often in the Ghanaian cohort than in the Malawian cohort.

One potential biological mechanism that could explain why higher iron status may be detrimental to the fetus is non–transferrin bound iron. Iron is typically carefully chaperoned around the body, predominately by transferrin. Unbound iron can result when the rate of iron influx into plasma exceeds the rate of iron acquisition by transferrin (40). It is therefore possible that higher iron status may lead to oxidative stress via unbound iron, which may result in lipid peroxidation and DNA damage of placental cells (41, 42) and impair the systemic response to infection (36), compromising the growth of the fetus. There is some evidence that a modest increase in plasma non–transferrin bound iron can occur after iron supplementation in nonpregnant women (40), although it is unclear whether this is relevant in our study population. It is unclear why IDA at enrollment in Ghana was associated with a lower risk of LBW, with iron deficiency at enrollment showing a similar nonsignificant trend.

Associations between iron status and birth outcomes may have differed by country owing to differences between Ghanaian and Malawian women in iron status at enrollment. Ghanaian women had higher iron status (mean sTfR: 4.1 compared with 5.6 mg/L; mean ZPP: 44.8 compared with 54.5 µmol/mol heme) than Malawian women at enrollment, and a lower proportion of Ghanaian women were identified as iron deficient (9.3% compared with 19.7%, sTfR >6 mg/L). All women in these 2 cohorts received iron supplementation, with approximately two-thirds receiving 20 mg and one-third receiving 60 mg Fe/d. Thus there was a larger number of iron-replete women in Ghana than in Malawi who received an amount of iron supplementation that may have been unnecessary, and plausibly had negative effects on the fetus. We previously demonstrated that birth weight was higher among infants born to women receiving SQ-LNSs (which contained 20 mg Fe) than among infants of women receiving iron and folic acid capsules (which contained 60 mg Fe), even though the iron and folic acid group had higher mean Hb concentration, higher iron status, and a lower prevalence of anemia at 36 weeks of gestation (18, 22). It is possible that there are other differences between the countries that might explain the different associations between iron status and birth outcomes.

Strengths of this study include the use of ultrasound to estimate gestational age at enrollment; the availability of a large number of covariates to test and control for confounding, including 2 markers of inflammation; and analysis of 2 cohorts from different regions in Africa where similar study methods were used. Including markers of inflammation is important, because inflammation can lead to elevated sTfR concentrations (43). In addition, we measured iron status during both early and late pregnancy. Concentrations of sTfR tend to be similar to nonpregnant concentrations during the first trimester, gradually increase during the second trimester, and peak in the third trimester (44).

We are limited in the interpretation of our results, because it is unclear whether low sTfR can be used as an indicator of iron-replete status. There is evidence that sTfR is lower during impaired erythropoiesis (39), which complicates the use of it as a marker of iron status. Further research to identify an accurate marker of iron-replete status during pregnancy is urgently needed. Other limitations include a delay in birth anthropometry for some infants, although back-calculations were performed according to WHO guidelines in such cases, as well as a possible limited generalizability of the findings in Malawi due to differences in some characteristics of included compared with excluded participants. However, differences between included and excluded participants were minimal. We tested multiple hypotheses but did not perform a statistical correction for multiple hypothesis testing because the birth outcomes are closely related to each other (45). Therefore, there is an increased risk that some findings could be due to chance. Measurements at 36 weeks of gestation do not include women who experienced a miscarriage or gave birth before 36 weeks, therefore survivor bias may affect the interpretation of associations with these measurements.

In conclusion, this research provides evidence that the associations between low or replete maternal iron status and birth outcomes are population specific. Future research to replicate and extend these findings would be beneficial.

## Supplementary Material

nxy278_Supplemental_Files

## Notes

Supported by Bill & Melinda Gates Foundation grant 49817 to the University of California, Davis and an Office of Health, Infectious Diseases, and Nutrition, Bureau for Global Health, US Agency for International Development (USAID) grant to the University of California, Davis under terms of Cooperative Agreement No. AID-OAA-A-12-00005 through the Food and Nutrition Technical Assistance III Project (FANTA), managed by FHI 360.

Author disclosures: BMO, JMJ, LMB, SA-A, KM, HO, JS, AL, PA, UA, SV, LHA, and KGD, no conflicts of interest.

Supplemental Methods, Supplemental Figure 1, and Supplemental Tables 1–9 are available from the “Supplementary data” link in the online posting of the article and from the same link in the online table of contents at https://academic.oup.com/jn/.

Abbreviations used: AGP, α-1-acid glycoprotein; CRP, C-reactive protein; Hb, hemoglobin; HCZ, head circumference *z* score; IDA, iron deficiency anemia; LAZ, length-for-age *z* score; LBW, low birth weight; PTB, preterm birth; PVE, plasma volume expansion; SGA, small-for-gestational-age; SQ-LNS, small-quantity lipid-based nutrient supplement; sTfR, soluble transferrin receptor; ZPP, zinc protoporphyrin.
