# Supplementary material for: Prenatal Iron Deficiency and Replete Iron Status Are Associated with Adverse Birth Outcomes, but Associations Differ in Ghana and Malawi
Source: J Nutr. 2019 Jan 9;149(3):513–21. doi: 10.1093/jn/nxy278 (PMC6398386; doi:10.1093/jn/nxy278)
Supplement: nxy278_Supplemental_Files [file nxy278_supplemental_files.zip › Oaks Fe status OSM figure 8_4_2018.pdf]

Supplementary Data

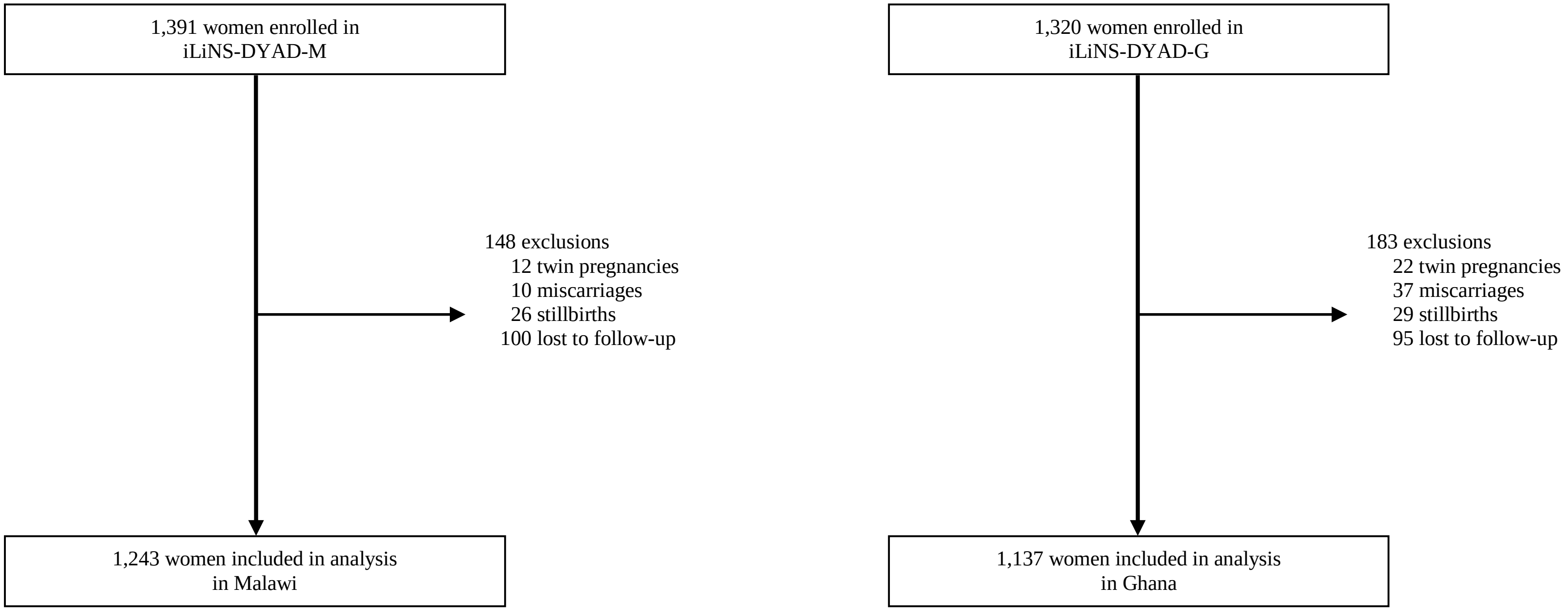

Supplemental Figure 1. Participant flowcharts for Malawi and Ghana study cohorts.
