# Supplementary material for: Prenatal Iron Deficiency and Replete Iron Status Are Associated with Adverse Birth Outcomes, but Associations Differ in Ghana and Malawi
Source: J Nutr. 2019 Jan 9;149(3):513–21. doi: 10.1093/jn/nxy278 (PMC6398386; doi:10.1093/jn/nxy278)
Supplement: nxy278_Supplemental_Files [file nxy278_supplemental_files.zip › Oaks Fe status OSM methods 10_3_2018.docx]

**Supplemental Methods**

Adjusted models for Table 3

**Adjusted models for Ghana**

1. Pregnancy duration: maternal BMI at enrollment, asset index, household food insecurity, infant sex, gestational age at enrollment, maternal age, maternal CRP at enrollment (baseline analyses only), maternal AGP and CRP at 36 wk (36 wk analyses only)
2. Birthweight: maternal BMI at enrollment, asset index, household food insecurity, infant sex, maternal age, parity, maternal AGP at 36 wk (36 wk analyses only)
3. Length-for-age z-score (LAZ): maternal BMI at enrollment, asset index, season at enrollment, maternal age, parity, maternal AGP at enrollment (baseline analyses only), maternal AGP and CRP at 36 wk (36 wk analyses only)
4. Head-circumference-for age z-score (HCZ): maternal BMI at enrollment, maternal age, parity, maternal AGP at 36 wk (36 wk analyses only)

**Adjusted models for Malawi:**

1. Pregnancy duration: gestational age at enrollment, maternal height, maternal CRP at enrollment (baseline analyses only), maternal CRP and AGP at 36 wk (36 wk analyses only), maternal malaria at enrollment, parity, season of enrollment, and site of enrollment
2. Birthweight: HIV status, maternal age, maternal height, maternal CRP and AGP at enrollment (baseline analyses only),maternal CRP and AGP at 36 wk (36 wk analyses only), maternal malaria at enrollment, parity, child sex, season of enrollment, and site of enrollment
3. LAZ: HIV status, maternal age, maternal height, maternal CRP and AGP at enrollment (baseline analyses only), maternal CRP and AGP at 36 wk (36 wk analyses only), maternal malaria at enrollment, parity, child sex, season of enrollment, and site of enrollment
4. HCZ: maternal age, maternal height, maternal CRP and AGP at enrollment (baseline analyses only), maternal CRP and AGP at 36 wk (36 wk analyses only), maternal malaria at enrollment, parity, season of enrollment, and site of enrollment

Adjusted models for Table 4

**Adjusted models for Ghana:**

1. PTB: maternal BMI at enrollment, asset index, household food insecurity, infant sex, gestational age at enrollment, maternal age, maternal CRP at enrollment (baseline analyses only), maternal AGP and CRP at 36 wk (36 wk analyses only)
2. LBW: maternal BMI at enrollment, asset index, household food insecurity, infant sex, maternal age, parity, maternal AGP at 36 wk (36 wk analyses only)
3. SGA: maternal BMI at enrollment, asset index, household food insecurity, infant sex, maternal age, parity, maternal AGP at 36 wk (36 wk analyses only)
4. Newborn stunting: maternal BMI at enrollment, asset index, season at enrollment, maternal age, parity, maternal AGP at enrollment (baseline analyses only), maternal AGP and CRP at 36 wk (36 wk analyses only)

**Adjusted models for Malawi:**

1. PTB: gestational age at enrollment, CRP and AGP at enrollment (baseline analyses only), AGP at 36 wk (36 wk analyses only), maternal malaria at enrollment, HIV status, parity, and site of enrollment
2. LBW: child sex, HIV status, maternal height, CRP and AGP at enrollment (baseline analyses only), parity, household food insecurity score, and site of enrollment
3. SGA: child sex, gestational age at enrollment, maternal AGP at baseline (baseline analyses only), maternal AGP at 36 wk (36 wk analyses only), maternal malaria at enrollment, HIV status, maternal age, maternal height, parity, season of enrollment, and site of enrollment
4. Newborn stunting: HIV status, maternal age, maternal height, maternal CRP and AGP at enrollment (baseline analyses only), maternal CRP and AGP at 36 wk (36 wk analyses only), maternal malaria at enrollment, parity, and site of enrollment

Adjusted models for Table 5

**Adjusted models for Ghana:**

1. PTB: maternal BMI at enrollment, asset index, household food insecurity, infant sex, gestational age at enrollment, maternal age, maternal CRP at enrollment (baseline analyses only), maternal AGP and CRP at 36 wk (36 wk analyses only)
2. LBW: maternal BMI at enrollment, asset index, household food insecurity, infant sex, maternal age, parity, maternal AGP at 36 wk (36 wk analyses only)
3. SGA: maternal BMI at enrollment, asset index, household food insecurity, infant sex, maternal age, parity, maternal AGP at 36 wk (36 wk analyses only)
4. Newborn stunting: maternal BMI at enrollment, asset index, season at enrollment, maternal age, parity, maternal AGP at enrollment (baseline analyses only), maternal AGP and CRP at 36 wk (36 wk analyses only)

**Adjusted models for Malawi:**

1. PTB: gestational age at enrollment, CRP and AGP at enrollment (baseline analyses only), AGP at 36 wk (36 wk analyses only), maternal malaria at enrollment, HIV status, parity, and site of enrollment
2. LBW: child sex, HIV status, maternal height, CRP and AGP at enrollment (baseline analyses only), parity, household food insecurity score, and site of enrollment
3. SGA: child sex, gestational age at enrollment, maternal AGP at baseline (baseline analyses only), maternal AGP at 36 wk (36 wk analyses only), maternal malaria at enrollment, HIV status, maternal age, maternal height, parity, season of enrollment, and site of enrollment
4. Newborn stunting: HIV status, maternal age, maternal height, maternal CRP and AGP at enrollment (baseline analyses only), maternal CRP and AGP at 36 wk (36 wk analyses only), maternal malaria at enrollment, parity, and site of enrollment

Adjusted models for Table 6

**Adjusted models for Ghana:**

1. PTB: maternal BMI at enrollment, asset index, household food insecurity, infant sex, gestational age at enrollment, maternal age, maternal CRP at enrollment (baseline analyses only), maternal AGP and CRP at 36 wk (36 wk analyses only)
2. LBW: maternal BMI at enrollment, asset index, household food insecurity, infant sex, maternal age, parity, maternal AGP at 36 wk (36 wk analyses only)
3. SGA: maternal BMI at enrollment, asset index, household food insecurity, infant sex, maternal age, parity, maternal AGP at 36 wk (36 wk analyses only)
4. Newborn stunting: maternal BMI at enrollment, asset index, season at enrollment, maternal age, parity, maternal AGP at enrollment (baseline analyses only), maternal AGP and CRP at 36 wk (36 wk analyses only)

**Adjusted models for Malawi:**

1. PTB: gestational age at enrollment, CRP and AGP at enrollment (baseline analyses only), AGP at 36 wk (36 wk analyses only), maternal malaria at enrollment, HIV status, parity, and site of enrollment
2. LBW: child sex, HIV status, maternal height, CRP and AGP at enrollment (baseline analyses only), parity, household food insecurity score, and site of enrollment
3. SGA: child sex, gestational age at enrollment, maternal AGP at baseline (baseline analyses only), maternal AGP at 36 wk (36 wk analyses only), maternal malaria at enrollment, HIV status, maternal age, maternal height, parity, season of enrollment, and site of enrollment
4. Newborn stunting: HIV status, maternal age, maternal height, maternal CRP and AGP at enrollment (baseline analyses only), maternal CRP and AGP at 36 wk (36 wk analyses only), maternal malaria at enrollment, parity, and site of enrollment

Adjusted models for Supplemental Table 3

**Adjusted models for Ghana:**

1. PTB: maternal BMI at enrollment, asset index, household food insecurity, infant sex, gestational age at enrollment, maternal age, maternal CRP at enrollment (baseline analyses only), maternal AGP and CRP at 36 wk (36 wk analyses only)
2. LBW: maternal BMI at enrollment, asset index, household food insecurity, infant sex, maternal age, parity, maternal AGP at 36 wk (36 wk analyses only)
3. SGA: maternal BMI at enrollment, asset index, household food insecurity, infant sex, maternal age, parity, maternal AGP at 36 wk (36 wk analyses only)
4. Newborn stunting: maternal BMI at enrollment, asset index, season at enrollment, maternal age, parity, maternal AGP at enrollment (baseline analyses only), maternal AGP and CRP at 36 wk (36 wk analyses only)

**Adjusted models for Malawi:**

1. PTB: gestational age at enrollment, CRP and AGP at enrollment (baseline analyses only), AGP at 36 wk (36 wk analyses only), maternal malaria at enrollment, HIV status, parity, and site of enrollment
2. LBW: child sex, HIV status, maternal height, CRP and AGP at enrollment (baseline analyses only), parity, household food insecurity score, and site of enrollment
3. SGA: child sex, gestational age at enrollment, maternal AGP at baseline (baseline analyses only), maternal AGP at 36 wk (36 wk analyses only), maternal malaria at enrollment, HIV status, maternal age, maternal height, parity, season of enrollment, and site of enrollment
4. Newborn stunting: HIV status, maternal age, maternal height, maternal CRP and AGP at enrollment (baseline analyses only), maternal CRP and AGP at 36 wk (36 wk analyses only), maternal malaria at enrollment, parity, and site of enrollment

Adjusted models for Supplemental Table 4

**Adjusted models for Ghana:**

1. PTB: maternal BMI at enrollment, asset index, household food insecurity, infant sex, gestational age at enrollment, maternal age, maternal CRP at enrollment (baseline analyses only), maternal AGP and CRP at 36 wk (36 wk analyses only)
2. LBW: maternal BMI at enrollment, asset index, household food insecurity, infant sex, maternal age, parity, maternal AGP at 36 wk (36 wk analyses only)
3. SGA: maternal BMI at enrollment, asset index, household food insecurity, infant sex, maternal age, parity, maternal AGP at 36 wk (36 wk analyses only)
4. Newborn stunting: maternal BMI at enrollment, asset index, season at enrollment, maternal age, parity, maternal AGP at enrollment (baseline analyses only), maternal AGP and CRP at 36 wk (36 wk analyses only)

**Adjusted models for Malawi:**

1. PTB: gestational age at enrollment, CRP and AGP at enrollment (baseline analyses only), AGP at 36 wk (36 wk analyses only), maternal malaria at enrollment, HIV status, parity, and site of enrollment
2. LBW: child sex, HIV status, maternal height, CRP and AGP at enrollment (baseline analyses only), parity, household food insecurity score, and site of enrollment
3. SGA: child sex, gestational age at enrollment, maternal AGP at baseline (baseline analyses only), maternal AGP at 36 wk (36 wk analyses only), maternal malaria at enrollment, HIV status, maternal age, maternal height, parity, season of enrollment, and site of enrollment
4. Newborn stunting: HIV status, maternal age, maternal height, maternal CRP and AGP at enrollment (baseline analyses only), maternal CRP and AGP at 36 wk (36 wk analyses only), maternal malaria at enrollment, parity, and site of enrollment

**NOTE:** All 36 wk models for Ghana and Malawi were additionally adjusted for intervention group.
