# Supplementary material for: Prenatal Iron Deficiency and Replete Iron Status Are Associated with Adverse Birth Outcomes, but Associations Differ in Ghana and Malawi
Source: J Nutr. 2019 Jan 9;149(3):513–21. doi: 10.1093/jn/nxy278 (PMC6398386; doi:10.1093/jn/nxy278)
Supplement: nxy278_Supplemental_Files [file nxy278_supplemental_files.zip › Oaks Fe status OSM tables 10_3_2018.docx]

Supplemental Table 1. Associations of maternal hemoglobin concentration and iron status with pregnancy outcomes in Ghana and Malawi.^1^

|  | **Pregnancy Duration** | | **Birth Weight** | | **LAZ** | | **HCZ** | |
| --- | --- | --- | --- | --- | --- | --- | --- | --- |
|  | Ghana | Malawi | Ghana | Malawi | Ghana | Malawi | Ghana | Malawi |
| **Early Pregnancy: ≤ 20 wk** |  |  |  |  |  |  |  |  |
| Hb |  | **+** |  | **+** |  | **+** |  |  |
| Higher iron status  (lower ZPP) |  | **+** |  |  | **(-)** | **(+)** |  |  |
| Higher iron status  (lower sTfR) |  | **+** |  | **+** | **-** | **+** |  | **+** |
| **Late Pregnancy: 36 wk** |  |  |  |  |  |  |  |  |
| Hb | Not linear |  |  |  |  |  |  |  |
| Higher iron status  (lower ZPP) |  | **(+)** | **-** |  | **-** |  |  | **(-)** |
| Higher iron status  (lower sTfR) |  | **(+)** | **-** |  | **-** |  | **-** |  |

^1^+: positive association, p <0.05; (+): marginally significant positive association, p <0.10; -: negative association, p<0.05; (-): marginally significant negative association, p<0.10; blank square: no association, p ≥ 0.10. Hb: hemoglobin; HCZ: head-circumference-for-age z-score; LAZ: length-for-age z-score; sTfR: soluble transferrin receptor; ZPP: zinc protoporphyrin.

Supplemental Table 2. Unadjusted associations of maternal hemoglobin, soluble transferrin receptor, and zinc protoporphyrin concentrations at ≤ 20 wk and 36 wk gestation with pregnancy duration and newborn anthropometric indicators.^1^

|  | **Hb** |  | **sTfR** | |  | **ZPP** | | |
| --- | --- | --- | --- | --- | --- | --- | --- | --- |
|  | Unadjusted β^2^ (95% CI) | | | Unadjusted β^2^ (95% CI) | | |  | Unadjusted β^2^ (95% CI) |
| **Early Pregnancy: ≤ 20 wk** |  | | |  | | |  |  |
| **Pregnancy duration** | | | |  | | |  |  |
| Ghana | 0.09 (0.03, 0.15) | | | 0.01 (-0.05, 0.07) | | |  | 0.03 (-0.03, 0.09) |
| Malawi | 0.12 (0.07, 0.18) | | | -0.09 (-0.15, -0.03) | | |  | -0.06 (-0.12, -0.01) |
| **Birth weight** |  | | |  | | |  |  |
| Ghana | 0.10 (0.04, 0.16) | | | 0.03 (-0.03, 0.09) | | |  | 0.01 (-0.05, 0.07) |
| Malawi | 0.12 (0.06, 0.18) | | | -0.11 (-0.18, -0.06) | | |  | -0.04 (-0.11, 0.02) |
| **Newborn LAZ** | | | |  | | |  |  |
| Ghana | 0.07 (0.02, 0.13) | | | 0.05 (0.00, 0.11) | | |  | 0.03 (-0.03, 0.09) |
| Malawi | 0.12 (0.06, 0.18) | | | -0.12 (-0.18, -0.06) | | |  | -0.06 (-0.12, 0.003) |
| **Newborn HCZ** | | | |  | | |  |  |
| Ghana | 0.06 (0.00, 0.12) | | | 0.04 (-0.02, 0.10) | | |  | 0.04 (-0.02, 0.10) |
| Malawi | 0.06 (0.004, 0.12) | | | -0.07 (-0.14, -0.01) | | |  | -0.04 (-0.10, 0.02) |
| **Late Pregnancy: 36 wk** |  | | |  | | |  |  |
| **Pregnancy duration** | | | |  | | |  |  |
| Ghana | --^3^ | | | 0.04 (-0.02, 0.07) | | |  | -0.01 (-0.06, 0.04) |
| Malawi | -0.02 (-0.04, 0.02) | | | -0.06, (-0.07, -0.001) | | |  | -0.08 (-0.08, -0.01) |
| **Birth weight** |  | | |  | | |  |  |
| Ghana | 0.02 (-0.04, 0.08) | | | 0.07 (0.01, 0.13) | | |  | 0.08 (0.02, 0.13) |
| Malawi | -0.001 (-0.06, 0.06) | | | -0.02 (-0.08. 0.04) | | |  | <0.01 (-0.06, 0.06) |
| **Newborn LAZ** | | | |  | | |  |  |
| Ghana | 0.00 (-0.06, 0.05) | | | 0.08 (0.02, 0.13) | | |  | 0.07 (0.00, 0.12) |
| Malawi | 0.03 (-0.04, 0.08) | | | -0.02 (-0.08, 0.04) | | |  | -0.01 (-0.07, 0.05) |
| **Newborn HCZ** | | | |  | | |  |  |
| Ghana | -0.01 (-0.07, 0.04) | | | 0.06 (0.00, 0.11) | | |  | 0.05 (-0.01, 0.10) |
| Malawi | -0.04 (-0.09, 0.03) | | | 0.02 (-0.04, 0.07) | | |  | 0.04 (-0.02, 0.10) |

^1^Standardized regression coefficients. Hb: hemoglobin; HCZ: head-circumference-for-age z-score; LAZ: length-for-age z-score; sTfR: soluble transferrin receptor; ZPP: zinc protoporphyrin.

^2^U-shaped relationship; see Figure 1.

Supplemental Table 3. Adjusted risk of adverse birth outcomes for women with anemia during early or late pregnancy.^1^

|  |  | **Reference group**  **(Hb 100-130) g/L**  **n/total n (%)** | **With anemia**  **(Hb <100 g/L)**  **n/total n (%)** | **Adjusted RR**  **(95% CI) ^2^** | **P** |
| --- | --- | --- | --- | --- | --- |
| **Early Pregnancy: ≤ 20 wk** | | |  |  |  |
| **Preterm birth** | |  |  |  |  |
| Ghana |  | 74/940 (7.9) | 21/165 (12.7) | 1.32 (0.81, 2.15) | 0.27 |
| Malawi |  | 71/881 (8.1) | 46/264 (17.4) | 1.72 (1.19, 2.50) | 0.004 |
| **Low birth weight** | | |  |  |  |
| Ghana |  | 109/939 (11.6) | 24/165 (14.6) | 0.93 (0.61, 1.42) | 0.74 |
| Malawi |  | 97/791 (12.3) | 35/219 (16.0) | 1.05 (0.74, 1.50) | 0.78 |
| **Small for gestational age** | | |  |  |  |
| Ghana |  | 195/910 (21.4) | 36/159 (22.6) | 0.94 (0.68, 1.30) | 0.70 |
| Malawi |  | 191/766 (24.9) | 62/213 (29.1) | 1.02 (0.80, 1.30) | 0.89 |
| **Newborn stunting** | | |  |  |  |
| Ghana |  | 87/935 (9.3) | 18/164 (11.0) | 0.90 (0.54, 1.50) | 0.69 |
| Malawi |  | 108/743 (14.5) | 53/213 (24.9) | 1.32 (0.98, 1.77) | 0.07 |
| **Late Pregnancy: 36 wk** | | |  |  |  |
| **Low birth weight** | | |  |  |  |
| Ghana |  | 71/825 (8.6) | 6/60 (10.0) | 0.64 (0.29, 1.42) | 0.27 |
| Malawi |  | 68/680 (10.0) | 20/194 (10.3) | 0.96 (0.59, 1.54) | 0.85 |
| **Small for gestational age** | | |  |  |  |
| Ghana |  | 183/803 (22.8) | 11/60 (18.3) | 0.58 (0.30, 1.10) | 0.09 |
| Malawi |  | 210/680 (30.9) | 48/192 (25.0) | 0.83 (0.63, 1.09) | 0.19 |
| **Newborn stunting** | | |  |  |  |
| Ghana |  | 44/822 (5.4) | 7/60 (11.7) | 1.19 (0.53, 2.65) | 0.67 |
| Malawi |  | 83/658 (12.6) | 31/187 (16.6) | 1.09 (0.74, 1.61) | 0.65 |

^1^ Preterm birth: <37 wk gestation; low birth weight: <2.5 kg; small for gestational age: birth weight <10^th^ percentile by gestational age and sex using the INTERGROWTH-21^st^ standard (30); stunting: length-for-age z-score < -2. Hb: hemoglobin.

^2^ RR: relative risk. Adjusted models included the following covariates if significant (p<0.1) with the outcome: gestational age at enrollment, parity, maternal age, education level, household food insecurity, household asset index, AGP at the time the blood sample was drawn, CRP at the time the blood sample was drawn, infant sex, maternal BMI at enrollment, maternal malaria at enrollment, and HIV status (Malawi models only). All 36 wk models adjusted for intervention group. Specifics regarding each adjusted model are provided in Supplemental Methods.

Supplemental Table 4. Adjusted risk of adverse birth outcomes for women with high hemoglobin status during early and late pregnancy.^1^

|  |  | **Reference Group**  **(Hb 100-130 g/L)**  **n/total n (%)** | **With high Hb**  **(Hb >130 g/L)**  **n/total n (%)** | **Adjusted RR**  **(95% CI)^2^** | **P** |
| --- | --- | --- | --- | --- | --- |
| **Early Pregnancy: ≤ 20 wk** | | |  |  |  |
| **Preterm birth** | |  |  |  |  |
| Ghana |  | 74/940 (7.9) | 3/54 (5.6) | 0.63 (0.20, 2.00) | 0.44 |
| Malawi |  | 71/881 (8.1) | 12/148 (8.1) | 1.13 (0.62, 2.05) | 0.70 |
| **Low birth weight** | | |  |  |  |
| Ghana |  | 109/939 (11.6) | 4/54 (7.4) | 0.74 (0.29, 1.85) | 0.51 |
| Malawi |  | 97/791 (12.3) | 14/133 (10.5) | 0.89 (0.53, 1.51) | 0.67 |
| **Small for gestational age** | | |  |  |  |
| Ghana |  | 195/910 (21.4) | 13/51 (25.5) | 1.27 (0.78, 2.06) | 0.34 |
| Malawi |  | 191/766 (24.9) | 26/130 (20.0) | 0.79 (0.54, 1.16) | 0.24 |
| **Newborn stunting** | | |  |  |  |
| Ghana |  | 87/935 (9.3) | 3/54 (5.6) | 0.64 (0.22, 1.92) | 0.43 |
| Malawi |  | 108/743 (14.5) | 13/131 (9.9) | 0.75 (0.43, 1.30) | 0.31 |
| **Late Pregnancy: 36 wk** | | |  |  |  |
| **Low birth weight** | | |  |  |  |
| Ghana |  | 71/825 (8.6) | 6/112 (5.4) | 0.68 (0.31, 1.51) | 0.35 |
| Malawi |  | 68/680 (10.0) | 11/84 (13.1) | 1.72 (0.94, 3.15) | 0.08 |
| **Small for gestational age** | | |  |  |  |
| Ghana |  | 183/803 (22.8) | 28/112 (25.0) | 1.08 (0.76, 1.53) | 0.68 |
| Malawi |  | 210/680 (30.9) | 22/84 (26.2) | 1.11 (0.77, 1.60) | 0.59 |
| **Newborn stunting** | | |  |  |  |
| Ghana |  | 44/822 (5.4) | 8/112 (7.1) | 1.29 (0.63, 2.64) | 0.50 |
| Malawi |  | 83/658 (12.6) | 12/82 (14.6) | 1.40 (0.80, 2.45) | 0.23 |

^1^Preterm birth: <37 wk gestation; low birth weight: <2.5 kg; small for gestational age: birth weight <10th percentile by gestational age and sex using the INTERGROWTH-21st standard (30); stunting: length-for-age z-score < -2. Hb: hemoglobin.

^2^RR: relative risk. Adjusted models included the following covariates if significant (p<0.1) with the outcome: gestational age at enrollment, parity, maternal age, education level, household food insecurity, household asset index, AGP at the time the blood sample was drawn, CRP at the time the blood sample was drawn, infant sex, maternal BMI at enrollment, maternal malaria at enrollment, and HIV status (Malawi models only). All 36 wk models adjusted for intervention group. Specifics regarding each adjusted model are provided in Supplemental Methods.

Supplemental Table 5. Unadjusted risk of adverse birth outcomes for women with anemia during early and late pregnancy.^1^

|  |  | **Reference group**  **Hb 100-130 g/L**  **n/total n (%)** | **With anemia**  **(Hb <100 g/L)**  **n/total n (%)** | **Unadjusted RR**  **(95% CI)^2^** |
| --- | --- | --- | --- | --- |
| **Early Pregnancy: ≤ 20 wk** | | |  |  |
| **Preterm birth** | |  |  |  |
| Ghana |  | 74/940 (7.9) | 21/165 (12.7) | 1.62 (1.03, 2.55) |
| Malawi |  | 71/881 (8.1) | 46/264 (17.4) | 2.16 (1.53, 3.05) |
| **Low birth weight** | | |  |  |
| Ghana |  | 109/939 (11.6) | 24/165 (14.6) | 1.25 (0.83, 1.89) |
| Malawi |  | 97/791 (12.3) | 35/219 (16.0) | 1.30 (0.91, 1.86) |
| **Small for gestational age** | | |  |  |
| Ghana |  | 195/910 (21.4) | 36/159 (22.6) | 1.06 (0.77, 1.45) |
| Malawi |  | 191/766 (24.9) | 62/213 (29.1) | 1.17 (0.92, 1.49) |
| **Newborn stunting** | | |  |  |
| Ghana |  | 87/935 (9.3) | 18/164 (11.0) | 1.18 (0.73, 1.91) |
| Malawi |  | 108/743 (14.5) | 53/213 (24.9) | 1.71 (1.27, 2.29) |
| **Late Pregnancy: 36 wk** | | |  |  |
| **Low birth weight** | | |  |  |
| Ghana |  | 71/825 (8.6) | 6/60 (10.0) | 1.16 (0.53, 2.56) |
| Malawi |  | 68/680 (10.0) | 20/194 (10.3) | 1.03 (0.64, 1.65) |
| **Small for gestational age** | | |  |  |
| Ghana |  | 183/803 (22.8) | 11/60 (18.3) | 0.80 (0.46, 1.39) |
| Malawi |  | 210/680 (30.9) | 48/192 (25.0) | 0.96 (0.73, 1.26) |
| **Newborn stunting** | | |  |  |
| Ghana |  | 44/822 (5.4) | 7/60 (11.7) | 2.18 (1.03, 4.63) |
| Malawi |  | 83/658 (12.6) | 31/187 (16.6) | 1.31 (0.90, 1.92) |

^1^Preterm birth: <37 wk gestation; low birth weight: <2.5 kg; small for gestational age: birth weight <10th percentile by gestational age and sex using the INTERGROWTH-21st standard (30); stunting: length-for-age z-score < -2. Hb: hemoglobin.

^2^RR: relative risk.

Supplemental Table 6. Unadjusted risk of adverse birth outcomes for women with iron deficiency during early and late pregnancy.^1^

|  |  | **Without Iron Deficiency**  **n/total n (%)^2^** | **With Iron Deficiency n/total n (%)^3^** | **Unadjusted RR**  **(95% CI)^4^** |
| --- | --- | --- | --- | --- |
| **Early Pregnancy: ≤ 20 wk** | | |  |  |
| **Preterm birth** | |  |  |  |
| Ghana |  | 77/924 (8.3) | 10/102 (9.8) | 1.18 (0.63, 2.20) |
| Malawi |  | 82/912 (9.0) | 41/248 (16.5) | 1.83 (1.30, 2.59) |
| **Low birth weight** | | |  |  |
| Ghana |  | 110/923 (11.9) | 7/102 (6.9) | 0.58 (0.28, 1.20) |
| Malawi |  | 100/813 (12.3) | 35/210 (16.7) | 1.36 (0.95, 1.93) |
| **Small for gestational age** | | |  |  |
| Ghana |  | 194/889 (21.8) | 19/100 (19.0) | 0.87 (0.57, 1.33) |
| Malawi |  | 195/788 (24.7) | 58/205 (28.3) | 1.14 (0.89, 1.47) |
| **Newborn stunting** | | |  |  |
| Ghana |  | 78/918 (8.5) | 7/102 (6.9) | 0.81 (0.38, 1.70) |
| Malawi |  | 114/762 (15.0) | 48/205 (23.4) | 1.57 (1.16, 2.11) |
| **Late Pregnancy: 36 wk** | | |  |  |
| **Low birth weight** | | |  |  |
| Ghana |  | 58/753 (7.7) | 10/141 (7.1) | 0.92 (0.48, 1.76) |
| Malawi |  | 53/560 (9.5) | 34/327 (10.4) | 1.10 (0.73, 1.65) |
| **Small for gestational age** | | |  |  |
| Ghana |  | 161/735 (21.9) | 28/138 (20.3) | 0.93 (0.65, 1.32) |
| Malawi |  | 137/546 (25.1) | 84/321 (26.2) | 1.04 (0.83, 1.32) |
| **Newborn stunting** | | |  |  |
| Ghana |  | 42/750 (5.6) | 6/141 (4.3) | 0.76 (0.33, 1.75) |
| Malawi |  | 76/536 (14.2) | 38/317 (12.0) | 0.85 (0.59, 1.22) |

^1^Preterm birth: <37 wk gestation; low birth weight: <2.5 kg; small for gestational age: birth weight <10th percentile by gestational age and sex using the INTERGROWTH-21st standard (30); stunting: length-for-age z-score < -2.

^2^Women with sTfR ≤6.0 mg/L. Reference group excluded women with high iron status (sTfR < 10^th^ percentile). At ≤ 20 wk, this was <2.49 mg/L for Ghana and <2.65 mg/L for Malawi. At 36 wk, this was <2.86 mg/L for Ghana and <3.08 mg/L for Malawi.

^3^sTfR >6 mg/L.

^4^RR: relative risk.

Supplemental Table 7. Unadjusted risk of adverse birth outcomes for women with iron deficiency anemia during early and late pregnancy.^1^

|  |  | **Without IDA**  **n/total n (%)^2^** | **With IDA**  **n/total n (%)^3^** | **Unadjusted RR**  **(95% CI)^4^** |
| --- | --- | --- | --- | --- |
| **Early Pregnancy: ≤ 20 wk** | | |  |  |
| **Preterm birth** | |  |  |  |
| Ghana |  | 92/1089 (8.5) | 5/48 (10.4) | 1.23 (0.53, 2.89) |
| Malawi |  | 104/1181 (8.8) | 25/106 (23.6) | 2.68 (1.82, 3.95) |
| **Low birth weight** | | |  |  |
| Ghana |  | 132/1088 (12.1) | 1/48 (2.1) | 0.17 (0.02, 1.20) |
| Malawi |  | 129/1051 (12.3) | 17/86 (19.8) | 1.61 (1.02, 2.54) |
| **Small for gestational age** | | |  |  |
| Ghana |  | 234/1051 (21.3) | 6/48 (12.5) | 0.56 (0.26, 1.20) |
| Malawi |  | 306/1051 (29.1) | 26/83 (31.3) | 1.27 (0.91, 1.78) |
| **Newborn stunting** | | |  |  |
| Ghana |  | 101/1083 (9.3) | 1/48 (2.1) | 0.22 (0.03, 1.57) |
| Malawi |  | 147/997 (14.7) | 25/85 (29.4) | 1.99 (1.39, 2.86) |
| **Late Pregnancy: 36 wk** | | |  |  |
| **Low birth weight** | | |  |  |
| Ghana |  | 81/963 (8.4) | 2/27 (7.4) | 0.88 (0.23, 3.40) |
| Malawi |  | 86/837 (10.3) | 10/102 (9.8) | 0.95 (0.51, 1.78) |
| **Small for gestational age** | | |  |  |
| Ghana |  | 217/941 (23.1) | 5/27 (18.5) | 0.80 (0.36, 1.79) |
| Malawi |  | 251/837 (30.0) | 25/101 (24.8) | 0.97 (0.68, 1.39) |
| **Newborn stunting** | | |  |  |
| Ghana |  | 57/960 (5.9) | 2/27 (7.4) | 1.25 (0.32, 4.85) |
| Malawi |  | 105/810 (13.0) | 16/95 (16.8) | 1.30 (0.80, 2.10) |

^1^Preterm birth: <37 wk gestation; low birth weight: <2.5 kg; small for gestational age: birth weight <10th percentile by gestational age and sex using the INTERGROWTH-21st standard (30); stunting: length-for-age z-score < -2. IDA: iron deficiency anemia.

^2^Women with Hb ≥ 100 g/L and sTfR ≤ 6.0 mg/L.

^3^Defined as Hb < 100 g/L and sTfR > 6.0 mg/L.

^4^RR: relative risk.

Supplemental Table 8. Unadjusted risk of adverse birth outcomes for women with high hemoglobin status during early and late pregnancy.^1^

|  |  | **Reference Group**  **Hb 100-130 g/L**  **n/total n (%)** | **With high Hb**  **(Hb >130 g/L)**  **n/total n (%)** | **Unadjusted RR**  **(95% CI)^2^** |
| --- | --- | --- | --- | --- |
| **Early Pregnancy: ≤ 20 wk** | | |  |  |
| **Preterm birth** | |  |  |  |
| Ghana |  | 74/940 (7.9) | 3/54 (5.6) | 0.71 (0.23, 2.17) |
| Malawi |  | 71/881 (8.1) | 12/148 (8.1) | 1.00 (0.56, 1.80) |
| **Low birth weight** | | |  |  |
| Ghana |  | 109/939 (11.6) | 4/54 (7.4) | 0.64 (0.24, 1.67) |
| Malawi |  | 97/791 (12.3) | 14/133 (10.5) | 0.86 (0.51, 1.45) |
| **Small for gestational age** | | |  |  |
| Ghana |  | 195/910 (21.4) | 13/51 (25.5) | 1.19 (0.73, 1.93) |
| Malawi |  | 191/766 (24.9) | 26/130 (20.0) | 0.80 (0.56, 1.16) |
| **Newborn stunting** | | |  |  |
| Ghana |  | 87/935 (9.3) | 3/54 (5.6) | 0.60 (0.20, 1.83) |
| Malawi |  | 108/743 (14.5) | 13/131 (9.9) | 0.67 (0.39, 1.17) |
| **Late Pregnancy: 36 wk** | | |  |  |
| **Low birth weight** | | |  |  |
| Ghana |  | 71/825 (8.6) | 6/112 (5.4) | 0.62 (0.28, 1.40) |
| Malawi |  | 68/680 (10.0) | 11/84 (13.1) | 1.31 (0.72, 2.37) |
| **Small for gestational age** | | |  |  |
| Ghana |  | 183/803 (22.8) | 28/112 (25.0) | 1.10 (0.78, 1.55) |
| Malawi |  | 210/680 (30.9) | 22/84 (26.2) | 1.00 (0.68, 1.47) |
| **Newborn stunting** | | |  |  |
| Ghana |  | 44/822 (5.4) | 8/112 (7.1) | 1.33 (0.65, 2.76) |
| Malawi |  | 83/658 (12.6) | 12/82 (14.6) | 1.16 (0.66, 2.03) |

^1^Preterm birth: <37 wk gestation; low birth weight: <2.5 kg; small for gestational age: birth weight <10th percentile by gestational age and sex using the INTERGROWTH-21st standard (30); stunting: length-for-age z-score < -2. Hb: hemoglobin.

^2^RR: relative risk.

Supplemental Table 9. Unadjusted risk of adverse birth outcomes for women with iron replete status during early or late pregnancy.^1^

|  |  | **Reference Group**  **(sTfR ≥ 10^th^ percentile) n/total n (%)^2^** | **With Iron Replete Status (sTfR <10^th^ percentile)**  **n/total n (%)^3^** | **Unadjusted RR**  **(95% CI)^4^** |
| --- | --- | --- | --- | --- |
| **Early Pregnancy: ≤ 20 wk** | | |  |  |
| **Preterm birth** | |  |  |  |
| Ghana |  | 77/924 (8.3) | 10/111 (9.0) | 1.08 (0.58, 2.03) |
| Malawi |  | 82/912 (9.0) | 6/128(4.7) | 0.53 (0.23, 1.18) |
| **Low birth weight** | | |  |  |
| Ghana |  | 110/923 (11.9) | 16/111 (14.4) | 1.21 (0.74, 1.97) |
| Malawi |  | 100/813 (12.3) | 11/115 (9.6) | 0.78 (0.43, 1.40) |
| **Small for gestational age** | | |  |  |
| Ghana |  | 194/889 (21.8) | 27/110 (24.6) | 1.12 (0.79, 1.60) |
| Malawi |  | 195/788 (24.7) | 24/111 (21.6) | 0.87 (0.60, 1.27) |
| **Newborn stunting** | | |  |  |
| Ghana |  | 78/918 (8.5) | 17/111 (15.3) | 1.80 (1.11, 2.93) |
| Malawi |  | 114/762 (15.0) | 10/116 (8.6) | 0.58 (0.31, 1.07) |
| **Late Pregnancy: 36 wk** | | |  |  |
| **Low birth weight** | | |  |  |
| Ghana |  | 58/753 (7.7) | 16/99 (16.2) | 2.10 (1.26, 3.50) |
| Malawi |  | 53/560 (9.5) | 14/99 (14.1) | 1.49 (0.86, 2.59) |
| **Small for gestational age** | | |  |  |
| Ghana |  | 161/735 (21.9) | 34/98 (34.7) | 1.58 (1.17, 2.15) |
| Malawi |  | 137/546 (25.1) | 23/98 (23.5) | 0.94 (0.64, 1.38) |
| **Newborn stunting** | | |  |  |
| Ghana |  | 42/750 (5.6) | 13/99 (13.1) | 2.34 (1.31, 4.21) |
| Malawi |  | 76/536 (14.2) | 12/101 (11.9) | 0.84 (0.47, 1.48) |

^1^Preterm birth: <37 wk gestation; low birth weight: <2.5 kg; small for gestational age: birth weight <10th percentile by gestational age and sex using the INTERGROWTH-21st standard (30); stunting: length-for-age z-score < -2.

^2^Reference group excluded women with iron deficiency (sTfR > 6.0 mg/L).

^3^sTfR <10^th^ percentile; at ≤ 20 wk, this was <2.49 mg/L for Ghana and <2.65 mg/L for Malawi. At 36 wk, this was <2.86 mg/L for Ghana and <3.08 mg/L for Malawi.

^4^RR: relative risk.
